# Chemical Ordering induced Strengthening in Lightweight Mg Alloys

**DOI:** 10.3390/nano12193488

**Published:** 2022-10-05

**Authors:** Yunnuo Duan, Qianfeng Gao, Zijian Zhang, Jiali Zhou, Yuze Li, Zongde Kou, Si Lan, Song Tang

**Affiliations:** 1 Herbert Gleiter Institute of Nanoscience, School of Materials Science and Engineering, Nanjing University of Science and Technology, Jiangsu 210094, China; 2 School of Physical Science and Technology, Northwestern Polytechnical University, Xi’an 710100, China

**Keywords:** Mg alloy, diffusion couple, phase transformation, ordered phases, precipitation strengthening

## Abstract

The influence of structure and composition on precipitation phenomena in Al-bearing BCC/HCP Mg alloys are studied via diffusion couple technique. Interdiffusion induced by the resultant composition gradient results in a change in crystal structure from HCP to BCC in the diffusion zone. The Vickers hardness in the diffusion zone is much higher than that in the Mg–5.5at.%Al and Mg–38at.%Li, which is attributed to the chemical ordering by nano-sized secondary ordered D0_3_–Mg_3_Al precipitation in the BCC Mg–Li–Al diffusion zone. The reasons for different precipitation in Al-bearing Mg alloys with various matrices are discussed. Generating ordered precipitates can be an effective approach to improve both strength and ductility in HCP Mg alloys.

## 1. Introduction

Mg–Li alloys with densities of 1.3–1.65 g/cm^3^ are attractive structural materials for lightweight applications in fields of automotive and aerospace to increase fuel efficiency and facilitate carbon reduction [1,2,3,4]. The matrix crystal structure configurations alters from hexagonal close-packed (HCP) to body-centered cubic (BCC) along with increased Li concentration in Mg–Li alloys. Specifically, when the Li content is less than 17.5 at.%, only the α (HCP solid solution) phase exists, and while the Li content is beyond 28.7 at.%, only the β (BCC solid solution) phase exists. Mixed α and β phases would appear in alloys for Li contents lying in between [5,6,7].

Recently, BCC β-based alloys are of high interest as they have a greater number of slip systems available compared with their α-based counterparts, which enables improved cold formability and further decreases in density [8]. Elemental Al is one main strengthening alloying element in BCC Mg–Li based alloys [9] and also the most common alloying element in conventional HCP Mg alloys [10]. It is necessary to clarify the role of concentration variations, especially Al, on structure and mechanical properties so as to guide the composition optimization and obtain high mechanical performance in HCP and/or BCC Mg alloys. Phase transformations in structural alloys are critical for creating suitable microstructures with desirable mechanical properties. It is not only time-consuming but also costly to explore the influences of structure and composition as fabrication of alloys with different compositions is often required. The diffusion couple technique is an efficient experimental approach to study phase transformations by virtue of the variations in chemistry and structure, leading to subsequent mechanical properties changes [11,12,13].

In this paper, the structure and composition dependence of precipitation phenomena in Mg alloys are investigated via the diffusion couple technique. Two model binary alloys, HCP Mg–5.5at.%Al and BCC Mg–38at.%Li, were prepared in the form of solid-to-solid diffusion couples to gain insight into their concentration-dependent interdiffusion-induced structural and corresponding mechanical properties variations.

## 2. Material and Methods

Mg–38at.%Li alloy and Mg–5.5at.%Al alloy were melted by high-purity pure metals (Mg, Li and Al) in an induction furnace with an argon-protected atmosphere and cast into a copper mold. The as-cast alloys were homogenized for 1 h at 350 ºC. Rectangular samples with dimensions of 10 mm × 10 mm × 6 mm were cut from the homogenized alloys for diffusion couple experiment. Mg–38at.%Li was coupled to Mg–5.5at.%Al through solid-to-solid diffusion couples to gain insight into the concentration-dependent interdiffusion of Al and Li in Mg and corresponding structural variations on mechanical properties.

**Figure 1:**
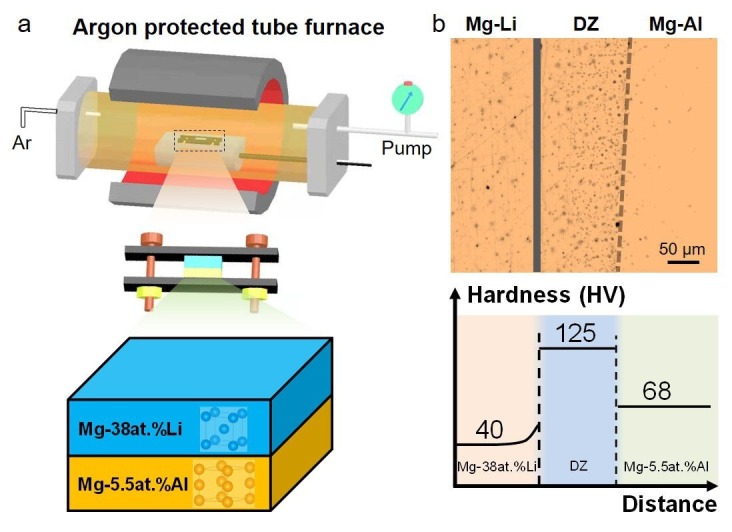
(**a**) Schematic illustration of the diffusion couple apparatus. (**b**) Optical micrograph and hardness profile of the Mg–38at.%Li/Mg–5.5at.%Al coupled sample. A clear diffusion zone (DZ) can be seen on the Mg–5.5at.%Al side after diffusion coupling. The Vickers hardness of Mg–38at.%Li and Mg–5.5at.%Al matrixes are 40 ± 2 HV and 68 ± 3 HV, and the hardness of the diffusion zone is 125 ± 5 HV. The mean value of hardness is displayed in the figure.

The serialized plates were prepared for diffusion bonding by sequentially polishing the surface with 3 μm, 1 μm and 0.5 μm oil-based polishing suspensions and mineral oil as a non-oxidizing lubricant. An intimate interface between two samples was achieved with the aid of a clamping jig. Constant and uniform clamping force could be achieved with stainless steel jigs owing to the much lower thermal expansion compared with experimental alloys. The assembly jig, shown in the middle of Figure 1a, consisted of two steel plates pulled together with two screws. The loaded jig was placed in a tube furnace with Ar protection (Figure 1a). The diffusion couple system was annealed for 60 h at 380 °C, at which temperature a single-phase solid solution was obtained for both alloys. After the annealing cycle, the loaded jig was quickly removed from the furnace followed by rapid cooling at ambient temperature to halt the diffusion process. The couple was extracted from the jig and cold-mounted in resin. Once cured, the couple was cross-sectioned and metallographically prepared down to a 0.5 μm finish. Particular attention had to be paid during metallographic sample preparation to keep the interdiffusion zone from debonding. Pure ethanol was used during the grinding and polishing processes, and polishing was performed with a lubricating oil.

X-ray diffraction (XRD) experiment was conducted to two separate alloys before coupling. The voltage and current of XRD experiments were 45 kV and 40 mA, respectively. The scanning resolution was 0.05° per step and scanning speed was 1° per min. Vickers hardness testing was carried out perpendicular to the diffusion-coupled surface by moving the indenter perpendicular to the joining surface during the test. The loads and loading time were chosen to be 0.01 kg and 15 s, respectively. Optical microscope (OM) and scanning electron microscope (SEM) observations were generated in the diffusion-coupled sample after polishing and etching with 5% Nital. The transmission electron microscope (TEM) samples were prepared by focused ion beam (FIB) with a gallium ion source. TEM experiments were conducted on an FEI Tecnai G^2^ T20 (FEI inc. Valley City, ND, USA) operated at 200 kV.

## 3. Results and Discussion

Figure 1b presents an optical micrograph and the hardness results of this diffusion-coupled sample. A diffusion zone with a width ~ 150 μm can clearly be seen at the Mg–5.5at.%Al side. Hardness testing was conducted on the diffusion-coupled sample. The results show that the Vickers hardness of Mg–38at.%Li and Mg–5.5at.%Al matrix are 40 ± 2 HV and 68 ± 3 HV, respectively, whereas the hardness of the diffusion zone is 125 ± 5 HV, which is much higher than that of either master alloy. This dramatic hardness increase is likely the result of interdiffusion related to possible phase transformations between the two alloys. This mechanical property difference prompted further exploration into the microstructural differences among the three zones.

**Figure 2:**
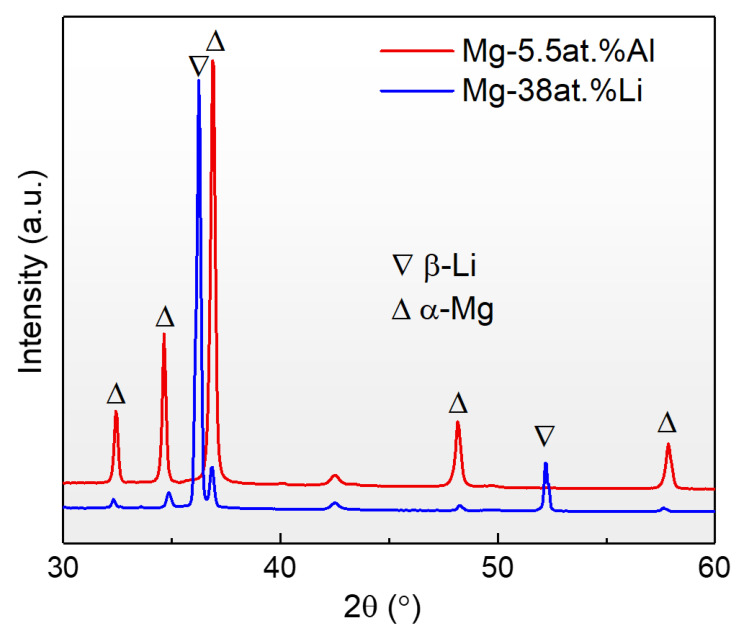
X-ray diffraction profile of solution-treated Mg–38at.%Li and Mg–5.5at.%Al. Mg–5.5at.%Al is made up of single HCP α phase, and a single BCC β matrix with minor α phase is achieved in Mg–38at.%Li.

**Figure 3:**
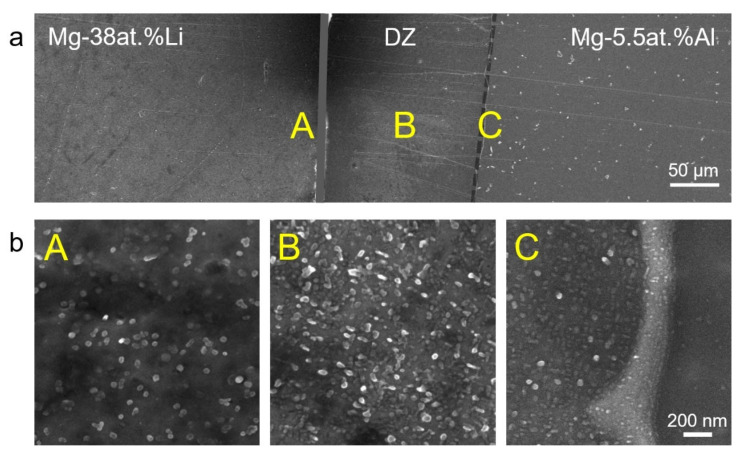
(**a**) SEM images of Mg–38at.%Li/Mg–5.5at.%Al diffusion couple. (**b**) Enlarged images of three areas A, B and C in (**a**). Nano-sized precipitates are observed in the diffusion zone, and some similar precipitates are found near the interface in Mg–38at.%Li.

Figure 2 displays the X-ray diffraction (XRD) results of solution-treated Mg–38at.%Li and Mg–5.5at.%Al before coupling. A single HCP α phase is obtained in Mg–5.5at.%Al, whereas a single BCC β matrix with minor α phase is observed in Mg–38at.%Li. No third phase was detected in either alloy. SEM images of the diffusion-coupled sample are shown in Figure 3. Of note are nano-sized third phase precipitates in the diffusion zone (marked “B”), which are similar in morphology to the θ’ precipitates in Al–Cu alloys. Owing to the interdiffusion of Al atoms, some similar precipitates with smaller size are found near the interface in Mg–38at.%Li (marked “A”). A clear interface between the diffusion zone and Mg–5.5at.%Al can be observed (marked “C”).

**Figure 4:**
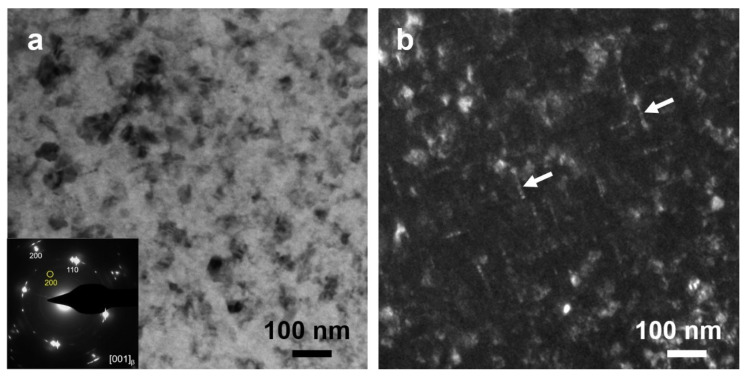
(**a**) Bright field TEM image in the diffusion zone. The inset shows SAED pattern from the [001]_β_ zone axis. The indexing for the matrix and superlattice spots are marked in white and yellow, respectively. (**b**) Dark-field TEM image obtained by electrons scattered by orientation from the yellow circle in (**a**). The superlattice spots correspond to the diffraction of rod-like D0_3_–Mg_3_Al precipitates (white arrows).

A detailed structural analysis of the rod-like precipitates in the diffusion zone was conducted by TEM, as shown in Figure 4. A typical bright-field TEM image (Figure 4a) shows a uniform distribution of nano-sized, rod-like precipitates. The selected area electron diffraction (SAED) pattern (Figure 4a inset) reveals a BCC β matrix and extra superlattice diffraction spots. The dark-field image (Figure 4b) taken from the spot bounded by the yellow circle demonstrates that the rod-like precipitates are superlattice phases, which are identified to be ordered D0_3_–Mg_3_Al, whose strengthening effect in BCC Mg–Li–Al has been elaborated elsewhere [14].

The composition gradient of Li, Al and Mg led to the interdiffusion between the two alloys. The matrix of the diffusion zone is converted to BCC from HCP owing to the Li content exceeding the threshold required to retain the HCP structure during the diffusion process. The strengthening Mg–Al intermetallic also forms as a consequence of phase transformations. From a quasi-chemical point of view, it is assumed that the heat of the mixing, Δ*H*_mix_, is caused only by the bond energies between adjacent atoms in a solution system [15]. Taking the binary system (containing A and B atoms) as an example, the change in internal energy (enthalpy) of the system during mixing is Δ*H*_mix_ = P_AB∙_ε, where P_AB_ is the number of A–B bonds, which is related to the number of bonds per atom and mole fractions of each atomic species; ε = ε_AB_-(ε_AA_ + ε_BB_)/2 is the difference between the A–B bond energy (ε_AB_) and the average of the A–A bond energy (ε_AA_) and B–B bond energy (ε_BB_). When the unlike bond energy is lower than average energy of the like bond, solid solutions have a negative enthalpy of mixing under this circumstance. It complies with our situation that atoms in both the BCC and HCP solid solution prefer unlike nearest neighbors and show a tendency to form Mg–Al intermetallic.

For the Mg–5.5at.%Al/Mg–38at.%Li diffusion couple, ordered D0_3_–Mg_3_Al is found to form within the BCC β matrix diffusion zone. However, the only phase that can precipitate in HCP Mg–5.5at.%Al during ageing is the Mg_17_Al_12_ intermetallic rather than an ordered phase. Considerations of electronegativity and strain lend some physical insights into formation of different Mg–Al phases. First, the electronegativities of Mg (1.31) and Al (1.61) differs by the Pauling scale [16], which drives formation of intermetallic compounds regardless of the matrix structure. Second, the atomic radii of Mg (1.60 Å) and Al (1.45 Å) differ by ~10%; thus, atomic radii difference induced mismatch strain in Mg–5.5at.%Al with close-packed structure (coordinating number of 12) is prone to be the major factor accounting for the formation complex Mg_17_Al_12_ cubic phase. For non-close-packed BCC structure (coordinating number of 8), the ordered D0_3_–Mg_3_Al phase can easily form as the activation energy barrier to nucleate ordered domains should be low because both the nucleus and matrix have essentially the same crystal structure and are close in composition; thus, the nuclei will be coherent with the low interfacial energy and strain energy to be overcome. Further investigations can be carried out by first-principles density functional theory calculations to examine the Gibbs free energy of different compounds in various matrices so as to design effective secondary strengthening phases with ordered structures.

It is intriguing to note that the observed precipitation phenomena indicate that the degree of precipitation strengthening induced by ordered-phase precipitation in BCC Mg–Li–Al alloys is much higher than that induced by complex-structured phase precipitation in HCP Mg–Al alloys. Indeed, ordered precipitates usually share certain coherency with the matrix, generating coherency hardening and order hardening [17,18]. The aforesaid strengthening modes are not applicable to secondary precipitates (such as Mg_17_Al_12_) that are incoherent with the matrix, and the precipitation hardening effects are much weaker in such alloy systems [19]. To date, there are no ordered precipitates experimentally reported in HCP Mg–Al alloys. Even so, via density functional theory, Hou et al. have already predicted the possible existence of an ordered D0_19_-Mg_3_Al phase that is more stable than other ordered structures [16]. This phase satisfies the metastability criteria and should be at least metastable. Furthermore, this phase is predicted to benefit the ductility in Mg-rich Mg–Al alloys. If ordered precipitates such as D0_19_-Mg_3_Al can be introduced into HCP Mg–Al alloys experimentally via some unique thermal–mechanical processing, a simultaneous improvement in strength and ductility in HCP Mg–Al alloys is believed to be achievable.

## 4. Conclusions

The effect of structure and composition on precipitation phenomena in binary Mg–Li and Mg–Al alloys were studied via Mg–5.5at.%Al/Mg–38at.%Li diffusion couple experiments. Interdiffusion induced by the resultant composition gradient resulted in a change in crystal structure from HCP to BCC in the diffusion zone. The Vickers hardness in the diffusion zone is much higher than that in Mg–5.5at.%Al and Mg–38at.%Li, which is attributed to the formation of the nano-sized secondary ordered D0_3_–Mg_3_Al precipitates. The atomic radii difference in Mg and Al induced mismatch strain in close-packed HCP Mg–Al alloys is prone to be the major factor accounting for the formation of complex-structured Mg_17_Al_12_ secondary phase. Generating nano-sized ordered precipitates in HCP Mg–Al alloys is believed to be beneficial to both strength and ductility.

## Data Availability

The raw/processed data required to reproduce these findings cannot be shared at this time as the data also form part of an ongoing study.
